# Medical Wards of St. Joseph’s Hospital, from May 1st to July 18th

**Published:** 1851-08

**Authors:** James Green

**Affiliations:** Resident Physician


*Medical Wards of St. Joseph's Hospital, from May 1st to July 18th.—Service of* Dr. W. V. Keating. Reported by James Green, M. D., Resident Physician.

| Disease. | Cured. | Relieved. | Died. | Removed. | Remaining. | Total. |
|---|---|---|---|---|---|---|
| Albuminuria - - | 0 | 0 | 1 | 0 | 0 | 1 |
| Amenorrhœa - | 3 | 0 | 0 | 0 | 2 | 5 |
| Anasarca - - | 0 | 0 | 1 | 0 | 0 | 1 |
| Anemia - - | 2 | 0 | 0 | 0 | 0 | 2 |
| Asthma - - | 0 | 1 | 0 | 0 | 0 | 1 |
| Bronchitis, acute - | 2 | 0 | 0 | 0 | 0 | 2 |
| " chronic | 1 | 1 | 0 | 0 | 1 | 3 |
| Cephalalgia - - | 1 | 0 | 0 | 0 | 0 | 1 |
| Cholera morbus - | 1 | 0 | 0 | 0 | 0 | 1 |
| Chorea - - - | 0 | 0 | 0 | 1 | 1 | 2 |
| Constipation - - | 1 | 0 | 1 | 0 | 0 | 2 |
| Delirium tremens - | 11 | 0 | 1 | 1 | 1 | 14 |
| Dysentery - - | 3 | 0 | 0 | 0 | 3 | 6 |
| Dyspepsia - - | 1 | 2 | 0 | 0 | 0 | 3 |
| Endocarditis - - | 0 | 0 | 0 | 1 | 0 | 1 |
| Erysipelas - - | 3 | 0 | 0 | 0 | 0 | 3 |
| Fever, intermittent | 2 | 0 | 0 | 0 | 0 | 2 |
| " remittent - | 0 | 0 | 0 | 0 | 1 | 1 |
| " typhoid - | 5 | 0 | 0 | 0 | 3 | 8 |
| " typhus - | 7 | 0 | 1 | 0 | 2 | 10 |
| Gastritis, acute - | 1 | 0 | 0 | 0 | 0 | 1 |
| Hysteria - - | 0 | 0 | 0 | 0 | 1 | 1 |
| Melancholia - - | 1 | 0 | 0 | 0 | 0 | 1 |
| Monomania -, - | 0 | 0 | 0 | 0 | 2 | 2 |
| Otorrhœa - - | 0 | 0 | 0 | 0 | 1 | 1 |
| Phthisis - - | 0 | 0 | 4 | 1 | 1 | 6 |
| Pleurisy - - | 2 | 0 | 0 | 0 | 0 | 2 |
| Pneumonia, acute - | 2 | 0 | 0 | 0 | 0 | 2 |
| " chronic | 3 | 0 | 0 | 0 | 1 | 4 |
| " pleuro | 1 | 0 | 0 | 0 | 1 | 2 |
| " typhoid | 0 | 0 | 1 | 0 | 2 | 3 |
| Procidentia - - |  | 0 | 0 | 0 | 1 | 1 |
| Prolapsus uteri - | 2 | 0 | 0 | 0 | 0 | 2 |
| Rheumatism - | 3 | 0 | 0 | 1 | 2 | 6 |
| Scarlatina - - | 2 | 0 | 0 | 0 | 0 | 2 |
| Spinal irritation - | 1 | 0 | 0 | 0 | 2 | 3 |
| Struck by lightning | 1 | 0 | 0 | 0 | 0 | 1 |
| Tonsillitis - - | 1 | 0 | 0 | 0 | 0 | 1 |
|  | 63 | 4 | 10 | 5 | 28 | 110 |

The case of constipation referred to in the report presents some points of interest; we therefore present the daily notes of the symptoms and treatment, together with the autopsy.

Ellen Carrigan, æt. 20, born in Ireland, been 3 years in this country, admitted into the St. Joseph's Hospital, June 1, 1851, about 5 P. M. Gave then the following history of her case:—

She had been living near the Schuylkill, and had suffered from intermittent fever, for which she had been treated and considered cured. At 5 o'clock in the evening of the day on which she was admitted, she woke up suffering extreme pain in the abdomen; medical aid was obtained, but finding comparatively no relief, she resolved to enter the Hospital.

When first seen by Dr. Green, the resident physician, she presented the following appearances:—Pulse about 100, not very small; skin hot and dry; tongue furred in the centre and slightly reddened at the edges and on the tip. Patient seemed to be in great pain; abdomen tympanitic and tender on pressure. Having ascertained that her bowels had not been opened for three days, Dr. Green administered copious enemata, and gave internally the following: Jalap Pulv. Hydg. C. mit. *aa*. gr. x.; this was repeated in two hours, the first having no effect. Pulv. opii gr. ss. was added to the above prescription the second time.

On Monday morning, June 2d, I saw Ellen for the first time. On entering the ward, I was struck with her appearance, her face giving evidence of extreme suffering. She complained of the violence of the pain in the abdomen, which after a careful examination I found to be seated in or near the transverse colon. Both this portion and the ascending and descending colon appeared hard and filled with impacted fæces. The abdomen was exceedingly tympanitic, but the extreme sensibility seemed confined to the region of the transverse colon. The pulse was about 100 and not very small; tongue slightly furred in the centre and red on the end and edges; papillæ slightly elevated; skin dry and harsh. The pain is paroxysmal, and she has intervals of almost entire relaxation from suffering, lasting from two to five minutes. Previous to the accession of pain, by placing the hand on the abdomen, we could feel the contractions of the abdominal muscles commencing, resembling very much the impression conveyed by placing the hand over the uterus in the first stage of labor. She vomited occasionally during her pains, a biliouslooking fluid, presenting nothing peculiar in its characters. Secretion of urine normal; no passage from the bowels.

I ordered cut cups over the abdomen, to be followed by a constant application of warm fomentations, the repetition of the enemata and pill; if these failed, the following: Hydg. C. mit. gr. v., Ol. Tiglii gtt. j., Opii Pulv. gr. j. M. in pilul. No. 1; if this did not produce the desired effect, to be repeated in three hours. She passed an uncomfortable day and night, her medicine having no effect, and the pain still retaining its paroxysmal character.

Tuesday morning, pulse the same as yesterday; skin jaundiced; no change in the tongue or skin; pain still continues; no passage from the bowels, but a profuse vomiting of fæcal matter took place this morning, whilst upon the close stool, passing an injection. I examined the abdomen carefully, and could not detect any hernia; abdomen still tympanitic, but not quite as tense or as tender as yesterday. She converses readily and seems quite easy during her intervals of pain; tongue natural, slightly furred in the centre; no appearance in the face of strangulation or invagination of the intestine, and if it had not been for the vomiting of fæcal matter, I would have unhesitatingly pronounced her something better. During all this time she complained of thirst, and was allowed lumps of ice.

Finding that the purgatives had no effect, I resolved to place her upon calomel and opium. I ordered, therefore, the following: Hydg. C. mit. gr. j., Dov. Pulv. gr. iv. q. h. 2, and a warm bath, to be followed up by warm fomentations.

Saw her Tuesday evening. Seems a good deal better, though still some pain; abdomen much relaxed since morning; vomiting ceased; pulse diminished in frequency; skin more moist; she seems quite cheerful and disposed to sleep. After an enema in the afternoon, passed a few scyballæ of a dark green color, but as yet no evacuation. I ordered an enema of castor oil f℥j, Croton oil gtt. ij, to be given at once, and the calomel and Dover's powder continued.

Wednesday morning. Found my patient much better; jaundiced hue of skin increased. She had passed a comfortable night; no vomiting; pain almost relieved, only a slight tenderness remaining over abdomen, which is entirely relaxed; had a profuse evacuation from the bowels, which consisted of a large mass of hardened scyballæ; urine free; pulse and tongue natural.

As she had some slight pain, I ordered her small doses of the solution of morphia, and an ordinary enema to be given.

Thursday morning. Ellen passed an excellent night; is quite cheerful; complains of a slight burning over the stomach; bowels open, stool natural, some pain in the uterine region; her period had arrived and she was menstruating. As she told me that she always suffered from dysmenorrhœa, I ordered a pill composed of 3 grs., of camphor and $\frac{1}{2}$ gr. of opium every two hours. She was permitted to get up, and I left her considering her convalescent.

All Thursday she seemed to improve; walked about the ward; seemed very cheerful. In the afternoon, whilst walking up the ward, she complained of sudden blindness and immediately expired.

*Post mortem.*—Heart and lungs perfectly healthy. Liver slightly enlarged. Gall bladder very much distended with bile. Large intestine perfectly healthy, save for two inches near the junction with ileum, where it presents a very dark appearance. The mucous membrane highly injected. The whole of the small intestines were of a dark mahogany color, and gave every indication of having been subjected to strangulation. About two inches above the ileo-cœcal valve I found a band constricting in such a manner as to include about eighteen inches of ileum. This band has a perfectly free border, and presents no indication of a rupture. It is twisted upon itself like a cord. The intestine is also twisted upon itself at this point. There is a small perforation in the ileum on the lower side of this band. It would seem as if the band had existed for a long time, and the constriction of the intestine had been caused by the twisting of this band upon itself. There was no effusion within the cavity of the peritoneum. The ileum around the perforation for about half an inch in circumference was much thinned, entirely denuded of its mucous coat, and appeared on the eve of perforation.

*Remarks.*—The above case presents some points of deep interest. The pain had ceased. A return of fæcal evacuations—and, in fine, the physique and morale of the patient afforded us every indication of perfect convalescence. And yet, without any of the prodromes of perforation, without any effusion in the cavity of the abdomen—the patient, as it were, in perfect convalescence—perishes from a disease which was stealthily progressing, without giving any manifestation of its onward course. We are at a loss to account for the suddenness of the death in any other manner, than by a powerful shock upon the great nervous centres, arresting the action of the heart. K.

*Surgical Wards of St. Joseph's Hospital, from May 1st to July 18th.—Service of* Dr. J. H. B. McClellan. Reported by R. Durney, M. D., Resident Physician.

